# The SPARK Study: a phase II randomized blinded controlled trial of the effect of furosemide in critically ill patients with early acute kidney injury

**DOI:** 10.1186/1745-6215-11-50

**Published:** 2010-05-11

**Authors:** Sean M Bagshaw, RT Noel Gibney, Finlay A McAlister, Rinaldo Bellomo

**Affiliations:** 1 Division of Critical Care Medicine, Faculty of Medicine and Dentistry, University of Alberta, 3C1.12 Walter C. Mackenzie Centre, 8440-112 Street, Edmonton, Alberta T6G 2B7, Canada; 2 Division of General Internal Medicine, Faculty of Medicine and Dentistry, University of Alberta, 2E3.24 Walter C. Mackenzie Centre, 8440-112 Street, Edmonton, Alberta T6G 2B7, Canada; 3 Department of Intensive Care, Austin Hospital, Studley Rd, Heidelberg, Victoria, 3084, Australia

## Abstract

**Background:**

Furosemide is commonly prescribed in critically ill patients with acute kidney injury (AKI). Existing data from observational studies and small clinical trials have significant limitations and have reported conflicting findings. There remains controversy on whether furosemide can impact clinical outcomes in critically ill patients with AKI; however, a survey of intensivists and nephrologists showed equipoise for high-quality evidence on this important issue.

**Design/Methods:**

This protocol summarizes the rationale and design of a phase II randomized, blinded, placebo-controlled trial of a low-dose continuous infusion of furosemide, titrated to the physiology parameter of urine output, in critically ill patients with early AKI. Two hundred sixteen adult critically ill patients with early evidence of AKI, defined by the RIFLE criteria, will be enrolled. Included patients will also have fulfilled ≥2 criteria of the systemic inflammatory response syndrome and achieved immediate goals of acute resuscitation. The primary outcome is progression in severity of kidney injury. Secondary outcomes include: safety, fluid balance, electrolyte balance, the need for renal replacement therapy, duration of AKI, rate of renal recovery, mortality and changes in novel serum and urine biomarkers of AKI. The primary analysis will be intention-to-treat. Planned recruitment will be complete by June 2011 and results available by December 2011.

**Trial Registration:**

ClinicalTrials.gov Identifier NCT00978354

## Background

Acute kidney injury (AKI) is common and increasingly encountered in hospitalized patients [1-3]. An estimated 6% of critically ill patients admitted to intensive care (ICU) develop severe AKI, and approximately 70% eventually receive acute renal replacement therapy (RRT)[4]. Moreover, critical illness complicated by AKI remains associated with high morbidity, mortality and health resource use [4-11].

There are few, if any, interventions proven to impact on the clinical course and outcome for critically ill patients once AKI is established[12,13]. However, important questions remain regarding the supportive role of selected interventions that still demand higher-quality evidence and better characterization in randomized trials to evaluate their impact in AKI. One example is: what is the role for loop diuretics, specifically furosemide, in the management of critically ill patients with early AKI?

Furosemide acts at the medullary thick ascending loop of Henle to inhibit the Na+/K+/Cl- pump on the luminal cell membrane surface and can theoretically reduce renal tubular oxygen demand[14,15]. Experimental data have also suggested low-dose furosemide infusion may attenuate ischemia/reperfusion-induced apoptosis and associated gene transcription in AKI[16,17]. These data support the hypothesis that the timely administration of furosemide may attenuate and/or reduce the severity of early onset AKI. In addition, furosemide may also have an important adjuvant role for maintaining fluid homeostasis and for optimal delivery of nutrition in critically ill AKI patients[18,19].

Furosemide remains the most common loop diuretic prescribed in critically ill patients[20]. In the BEST Kidney Study, a large multi-centre observational study of >1700 critically ill patients with AKI, 70% had received diuretics at the time of enrollment, of whom 98% were receiving furosemide[21]. While numerous studies have evaluated loop diuretics in the treatment of AKI [22-30], the majority have failed to find consistent clinical benefit. Moreover, two large observational studies of AKI in critically ill patients have reported discrepant findings on the effect of loop diuretics on mortality and renal recovery[21,31]. Additional small trials have suggested that diuretics may reduce the severity of kidney injury by converting "oliguric" to "non-oliguric" AKI, shorten the duration of AKI, improve the rate of renal recovery, and perhaps delay or ameliorate need for RRT [22,24,32-35]. However, improvements in survival or renal recovery have yet to be confirmed with high-quality evidence. Accordingly, there is controversy as to whether furosemide can impact clinical outcomes and should be used in critically ill patients with AKI [36-40].

A recent systematic review of randomized trials assessing the role of loop diuretics in AKI found five trials enrolling 555 patients that focused on critically ill patients[41]. This review found no statistical difference in mortality (odds ratio [OR], 1.28, p = 0.18) or renal recovery (OR, 0.88, p = 0.5) for use of loop diuretics compared with control. However, loop diuretics were associated with a shorter duration of RRT (weighted mean difference, -1.4 days, p = 0.02), shorter time to spontaneous decline in serum creatinine (weighted mean difference, -2.1 days, p = 0.01) and a greater increase in urine output from baseline (OR, 2.6, p = 0.004). There was insufficient data to comment on the impact of loop diuretics on electrolyte abnormalities, fluid balance, duration of mechanical ventilation, secondary organ dysfunction, hospital length of stay or health costs.

Importantly, however, this review found that the overall trial quality and applicability of this evidence to critically ill patients was poor[41]. For example, trials were generally small, confounded by co-interventions (i.e. mannitol, dopamine), and characterized by delayed or late intervention (i.e. prolonged periods of oligo-anuria or already receiving RRT at the time of enrollment). Finally, these trials often administered furosemide as large intravenous bolus doses with no specific titration of therapy to physiologic endpoints such as urine output. As a consequence, these data have limited applicability to modern critically ill patients and to current ICU practice.

A recent multi-national survey of intensivists and nephrologists showed that most respondents did not believe that furosemide use in AKI would directly improve outcomes such as mortality, need for RRT, or renal recovery[20]. However, there was significant uncertainty about the existing evidence and the majority had equipoise for and supported a trial on this issue. Accordingly, we have proposed a phase II randomized, blinded, placebo-controlled trial of a furosemide infusion titrated to urine output in critically ill patients with early AKI.

## Objectives

The specific objectives of this trial are:

• To compare the efficacy and safety of a continuous infusion of furosemide versus placebo titrated to the physiology parameter of urine output in early AKI on the primary outcome of progression in severity of kidney injury from early AKI.

• To evaluate the impact of furosemide versus placebo on key secondary endpoints including: fluid balance; electrolyte and acid-base balance; the need for RRT; total duration of AKI; the rate of renal recovery; and mortality.

• To evaluate the impact of furosemide versus placebo on the tertiary endpoint of differences, trajectory and prognostic value of novel serum and urine biomarkers for AKI.

## Design/Methods

### Study Design, Setting and Patient Population

This is a phase II randomized, blinded, placebo-controlled trial of ICU patients with early AKI with randomization stratified by sepsis. This study will be performed at the University of Alberta Hospital (UAH) General Systems Intensive Care Unit (GSICU). The UAH is an academic/tertiary care hospital and regional trauma centre with approximately 1300-1400 annual admissions. All patients admitted to the GSICU will be screened for eligibility.

### Operational Definitions

#### Acute kidney injury (AKI)

The operational definition for early AKI will be defined and classified according to a modified RIFLE criteria (acronym indicating Risk of renal dysfunction; Injury to the kidney; Failure of kidney function; Loss of kidney function; and End-stage kidney disease) as outlined by the ADQI Working Group [42]. In brief, the RIFLE criteria classifies AKI into three categories of severity (Risk, Injury, and Failure) and two categories of clinical outcome (Loss and End-stage kidney disease) based on relative changes to serum creatinine and urine output. The presence of early AKI will be defined by a minimum of *RIFLE class - RISK*: an abrupt (within 7 day) reduction in kidney function characterized by an relative increase in serum creatinine of ≥ 50% (1.5 fold) or an absolute increase of ≥ 26.5 μmol/L from baseline or a reduction in urine output of ≤ 0.5 mL/kg/hr for ≥ 6 hours. For reference, the RIFLE category INJURY is defined as a relative increase in serum creatinine ≥ 100% (2.0 fold) or a reduction in urine output of ≤ 0.5 mL/kg/hr for ≥ 12 hours. The RIFLE category FAILURE is defined as a relative increase in serum creatinine ≥ 200% (3.0 fold) or an absolute value ≥354 μmol/L (accompanied by an acute increase ≥44.2 μmol/L) or a reduction in urine output of ≤0.3 mL/kg/hr for ≥ 24 hours or anuria for ≥ 12 hours.

#### Renal replacement therapy (RRT)

The operational definition of RRT will incorporate any form of extracorporeal renal support or replacement for patients with documented AKI. By protocol and in order to minimize the potential bias of clinician discretion on when to initiate RRT, at least one of the following criteria must be fulfilled prior to initiation of RRT: 1) refractory oliguria (urine output <100 mL in preceding 4 hrs, despite fluid resuscitation and/or vasoactive therapy F OR maximum dose of study drug); 2) refractory extravascular fluid overload AND/OR hypoxemia AND/OR pulmonary edema (FiO2 ≥60%, receiving mechanical ventilation, PaO2/FiO2 ratio ≤200); 3) azotemia (urea ≥30 mmol/L); 4) metabolic acidosis (pH <7.2 or HCO3 <15); hyperkalemia ([K^+^] ≥6.0 mmol/L or electrocardiogram changes, despite maximum dose of study drug AND/OR administration of at least 1 dose of potassium binder AND/OR intravenous insulin AND/OR intravenous bicarbonate; 5) uremia-induced organ toxicity (i.e. encephalopathy, pericarditis).

#### Renal recovery

The operational definition of renal recovery will be the return of serum creatinine to within 10% of baseline levels and a spontaneous urine output ≥1.0 mL/kg/hr for a minimum of 24 hours independent of RRT.

#### Systemic inflammatory response syndrome (SIRS)

The SIRS criteria include the presence of any 2 of the following: temperature >38°C or <36°C; heart rate >90 beats/min; respiratory rate >20 breaths/min or PaCO_2 _<32 mmHg or mechanically ventilated; and/or white cell count >12,000 cells/mm^3^, <4,000 cells/mm^3 ^or with >10% immature (band) forms[43].

#### Sepsis

The operational definition of the clinical syndrome of sepsis will be the presence of confirmed or suspected infection and the presence of ≥ 2 SIRS criteria.

### Inclusion Criteria

Patients must fulfill all of the following inclusion criteria:

• Peripheral or central intravenous catheter and urinary catheter

• Early AKI

• ≥ 2 criteria for the systemic inflammatory response syndrome (SIRS) within 24 hours

• Achieved immediate resuscitation goals (as directed by the treating physician) including fluid resuscitation AND/OR vasoactive therapy to achieve mean arterial pressure ≥65 mmHg, central venous pressure ≥8 cmH2O, central venous oxygen saturation ≥70% (if measured) AND/OR cardiac index ≥2.5 L/min/1.73 m^2 ^(if measured).

### Exclusion criteria

The following conditions will lead to ineligibility for study entry:

• Age <18 years

• Confirmed or suspected pregnancy (verified by serum [β-HCG] pregnancy test if necessary)

• Obstructive etiology for AKI

• ≥ Stage 4 chronic kidney disease (defined by an estimated glomerular filtration rate <30 mL/min/1.73 m^2^), end stage kidney disease on chronic RRT, kidney transplantation or already received RRT in ICU

• Resolving AKI, defined as a ≥25% or ≥44.2 μmol/L decline from peak increase in serum creatinine

• Acute pulmonary edema requiring urgent use of furosemide or RRT or patient already receiving continuous furosemide infusion

• Patient is moribund with expected death within 24 hours

• Known or suspected drug allergy to furosemide

• Enrolled in concomitant randomized trial

• Prior enrollment in SPARK

### Trial Protocol

#### Description of Study Flow

**Figure 1:**
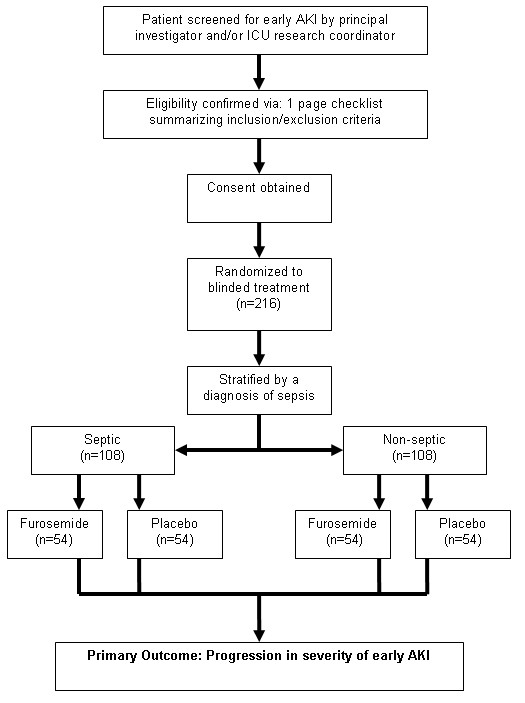
**Overview of patient flow**.

Figure 1 outlines the study flow. Patients will be identified in the ICU by daily surveillance by the research coordinator or when identified by the treating ICU physician. Each patient's eligibility will be verified by use of a one-page checklist that summarizes the inclusion and exclusion criteria. This checklist will be included in the standardized case-report form (CRF).

### Study Intervention

**Figure 2:**
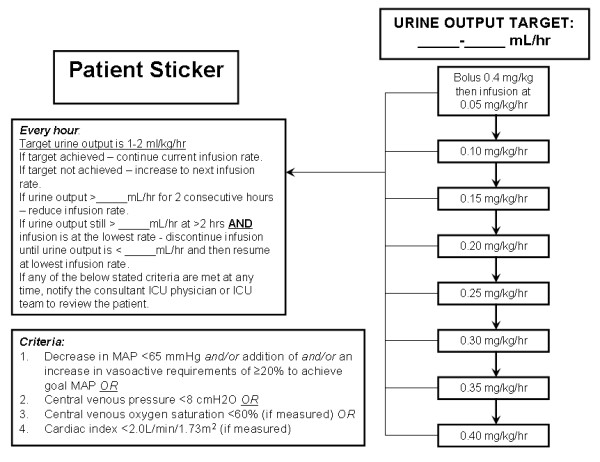
**Overview of the SPARK study drug infusion algorithm**.

Patients will be commenced on a continuous infusion of either the intervention (furosemide) or identical placebo (0.9% NaCl). The study protocol for administration of furosemide by continuous infusion is adapted from the phase I study by Ostermann et al [44] (Figure 2).

The study infusion bag will contain 2000 mg of furosemide in 500 mL of 0.9%NaCl for a final concentration of 4 mg/mL. The appearance of the study infusion bags for furosemide and placebo will be identical and there will be no marking on the infusion bags other than a 4-digit coded identifying study number. This will minimize bias and ensure blinding of allocation by the study investigators, research coordinator, ICU physicians and all involved health care providers.

The continuous infusion will be titrated to achieve a target urine output in the range of 1.0-2.0 mL/kg/hr. The treatment algorithm for titration of the continuous infusion is shown in Figure 2. Each patient will be administered a loading dose of 0.4 mg/kg as a separate infusion bag followed by a continuous infusion commenced at a dose of 0.05 mg/kg/hr (Table 1). The maximum infusion rate will be 0.40 mg/kg/hr. The urine output will be assessed hourly. If the target urine output has been achieved, then the current infusion rate will be continued. If the target urine output has not been achieved, the dose will be increased to the next infusion rate in the algorithm. If the urine output is too brisk (>2 mL/kg/hr x 2 hrs), the infusion rate will be reduced. If the urine output is still >2 mL/kg/hr AND the infusion rate is at the lowest rate, the study drug will be discontinued infusion until the urine output is <1 mL/kg/hr. If any of the following criteria are fulfilled: decrease in mean arterial pressure <65 AND/OR addition of or an increase in vasoactive requirements of ≥20% to achieve goal mean arterial pressure OR central venous pressure <8 cmH2O OR central venous oxygen saturation <60% OR a cardiac index <2.0 L/min/1.73 m^2 ^(if measured) at any time, the consultant ICU physician and ICU team will be notified to review the patient.

**Table 1:** Summary of weight-based categories for commencement for study infusion rate (concentration study drug 4 mg/mL)

Weight Category (kg)	Bolus loading dose	Start infusion dose	Start infusion dose (mg/hr)	Start infusion rate (mL/hr)
≤50	0.4 mg/kg	0.05 mg/kg/hr	2.5	0.6

55	0.4 mg/kg	0.05 mg/kg/hr	2.8	0.7

60	0.4 mg/kg	0.05 mg/kg/hr	3.0	0.8

65	0.4 mg/kg	0.05 mg/kg/hr	3.3	0.8

70	0.4 mg/kg	0.05 mg/kg/hr	3.5	0.9

75	0.4 mg/kg	0.05 mg/kg/hr	3.8	0.9

80	0.4 mg/kg	0.05 mg/kg/hr	4.0	1.0

85	0.4 mg/kg	0.05 mg/kg/hr	4.3	1.1

90	0.4 mg/kg	0.05 mg/kg/hr	4.5	1.1

≥95	0.4 mg/kg	0.05 mg/kg/hr	4.8	1.2

An estimate of patient ideal body weight (IBW) will be used to determine the urine output target. Determination of estimated IBW will be based on the formula described by Devine[45] (Table 2). From this estimated IBW, patients will be divided into 5 kg weight categories to determine urine output goals for protocol simplicity (Table 3). At any time during the trial, if the responsible ICU physician believes that the administration of furosemide is urgently indicated (i.e. new pulmonary edema), it can be administered and this event will be documented.

**Table 2:** Devine formulate for ideal body weight estimation[45].

Sex	Devine Formula				
Female	IBW (kg) = [45 +(0.91 x (height in cm - 152))]				

Male	IBW (kg) = [50+ (0.91 x (height in cm - 152))]				

					

**Height (cm)**	**Height (feet)**	**Female**		**Male**	
		
		**IBW (kg)**	**Category**	**IBW (kg)**	**Category**

≤152	≤5'0	45	≤50	50	≤50

155	5'1	47.7	≤50	52.7	55

157	5'2	49.6	≤50	54.6	55

160	5'3	52.3	55	57.3	60

163	5'4	55.0	55	60.0	60

165	5'5	56.8	60	61.8	65

168	5'6	59.6	60	64.6	65

170	5'7	61.4	65	66.4	70

173	5'8	64.1	65	69.1	70

175	5'9	65.9	70	70.9	75

178	5'10	68.7	70	73.7	75

180	5'11	70.5	75	75.5	80

183	6'0	73.2	75	78.2	80

185	6'1	75.0	75	80.0	80

188	6'2	77.8	80	82.8	85

191	6'3	80.5	85	85.5	90

193	6'4	82.3	85	87.3	90

196	6'5	85.0	85	90.0	90

≥ 198	≥ 6'6	86.9	90	91.9	95

**Table 3:** Summary of weight-based urine output targets.

Weight Category (kg)	Target Urine Output (mL)			
	
	Hourly	Per 6 hours	Per 12 hours	Per 24 hours
≤50	50-100	300-600	600-1200	≥ 1200

55	55-110	330-660	660-1320	≥ 1320

60	60-120	360-720	720-1440	≥ 1440

65	65-130	390-780	780-1560	≥ 1560

70	70-140	420-840	840-1680	≥ 1680

75	75-150	450-900	900-1800	≥ 1800

80	80-160	480-960	960-1920	≥ 1920

85	85-170	510-1020	1020-2040	≥ 2040

90	90-180	540-1080	1080-2160	≥ 2160

≥ 95	95-190	570-1140	1140-2280	≥ 2280

All other aspects of patient management within the parameters outlined (i.e. methods of fluid resuscitation, choice of fluids, vasoactive therapy, choice of vasoactive therapy, adjuvant therapies such as hrAPC, intensive insulin therapy) will be at the discretion of the consultant ICU physician. No other interventions will be performed.

The study drug infusion will be continued for a minimum of 24 hrs and discontinued if any one of the following events occurs:

• The patient is initiated on RRT;

• The patient is discharged from the ICU;

• The patient recovers kidney function;

• The patient dies;

• The patient develops a recognized adverse reaction potentially related to the study infusion; or

• The patient has received a total of 7-days of study drug administration.

### Methods of Randomization, Concealment and Blinding

The randomization sequence will be created at a single central location at the Epidemiology and Research Coordinating Centre (EPICORE) at the University of Alberta (available at: http://www.epicore.ualberta.ca/index.html). This randomization sequence will be stratified by the presence of a diagnosis of sepsis. The clinical trial pharmacist (unblinded) will use a web-based randomization program to determine allocation of patients and then prepare the coded study solution. Each coded study solution bag will then be dispensed for administration to the patient as per protocol. This coded identifying study number will also be labeled on the patient CRF. The investigators, study coordinators, treating physicians, bedside nurses and patients/family will remain blinded to the allocated study solution.

### Data Collection

Detailed clinical, procedure-related, physiologic and laboratory data will be collected. Blood and urine will be collected at baseline, 12 hours, 24 hours and daily thereafter until participants exits the study. Data will be collected on standardized CRFs developed by the EPICORE centre. Completed CRF will be returned to the EPICORE centre, entered into a central database, where data queries will be generated.

Clinical data captured will include demographics, co-morbidities and prescribed/current drug therapy. Details of admission diagnoses, surgical status, and dates of hospital and ICU admission will be recorded. Detailed data will be recorded on date of enrollment (i.e. fulfilling criteria for early AKI). This will include details of interventions (i.e. mechanical ventilation, vasoactive drugs, fluid therapy), hemodynamics (i.e. blood pressure, heart rate, central venous pressure), and acute physiology (i.e. components of severity of illness scores, urine output, fluid balance, secondary non-kidney organ dysfunction). During the trial, data will be collected daily on urine output, fluid balance, electrolytes, acid-base status, serum creatinine and urea. Collected blood and urine samples will be stored for batched analysis of kidney-injury specific biomarkers, including: serum and urine cystatin C, serum and urine neutrophil gelatinase-associated lipocalin (NGAL), urine interleukin-18 (IL-18); urine kidney injury molecule-1 (KIM-1), and urine L-type fatty acid binding protein (L-FABP).

Data will be collected each day on whether the primary endpoint (progression of AKI) has occurred, for evidence of any secondary endpoints and for criteria for trial discontinuation.

Finally, any study protocol violations will be recorded. The adjudication of protocol violations will be determined by a study investigator blinded to the treatment allocation.

All enrolled patients will be followed to determine the duration of AKI, continued need for RRT, renal recovery and mortality until death or discharge from hospital and at 30, 60 and 90-days after randomization.

### Sample Size Estimation

Our primary outcome measure for this trial is progression from early AKI (RIFLE class - **RISK**) to a more severe form of AKI, defined by progression in AKI to the development of either RIFLE class - **Injury or Failure**. Based on data from a large observational study, an estimated 60% of critically ill patients with early AKI worsen and develop further kidney injury[46]. We estimate that furosemide will contribute to a 20% absolute reduction in the proportion of those patients who progress from early AKI (RIFLE class - **RISK**) to either RIFLE class - **Injury or Failure**. This would require a total sample size of 214 patients and provide 80% power (alpha 0.05) for detection of a 20% difference in the proportion with progression of AKI. An estimated 50% of patients developing early AKI will have sepsis[47]. Randomization will be stratified by the presence of sepsis. The total sample size was increased to 216 patients to ensure a balanced number of patients in each treatment arm (Figure 1).

### Statistical Analysis

The primary analysis will evaluate the proportion of critically ill patients with early AKI that have progression in kidney injury by having received furosemide or placebo. Analysis will be intention-to-treat.

The secondary analysis will evaluate for differences between furosemide and placebo in cumulative fluid balance and in the largest changes to serum potassium, serum magnesium, serum pH and serum bicarbonate levels, defined as difference from enrollment to lowest documented level during study infusion. In addition, this secondary analysis will evaluate for differences in the need for RRT, duration of AKI, rate of renal recovery and hospital mortality.

Descriptive statistics, boxplots and histograms will be used to analyze individual baseline variables by having received furosemide or placebo. Normally or near normally distributed, non-correlated variables will be reported as means with standard deviations (SD) and compared using the appropriate Student's t test. Non-normally distributed, non-correlated continuous data will be reported as medians with inter-quartile ranges (IQR) and compared using the Mann Whitney U test. Non-correlated categorical data, including the primary outcome, and need for RRT, renal recovery and hospital mortality, will be reported as proportions and compared using Fisher's Exact Test. If necessary, multi-variable logistic or linear regression will be used to control for potential confounding from imbalances in baseline characteristics after randomization.

The tertiary analysis will evaluate for differences between furosemide and placebo in the change of novel serum and urine biomarkers for AKI, defined by absolute levels and relative to baseline (enrollment) at 12 hours, 24 hours and at the time of initiation of RRT, renal recovery or ICU discharge. All samples will remain blinded during processing, storage and analysis. Normally distributed correlated data will be analyzed by the repeated measures ANOVA. Non-normally distributed correlated data will be analyzed by the Friedman test. Correlated categorical data will analyzed by generalized estimating equations. The diagnostic and predictive characteristics of absolute values and relative changes in novel serum and urine biomarkers for progression of AKI as well as need for RRT will be evaluated by 2 x 2 tables and construction of receiver operator characteristic curves. A p-value of < 0.05 will be considered significant. All statistical tests will be two-sided.

### Data Safety and Monitoring

The trial will have a data safety and monitoring committee (DSMC) that will consist of four members. Three members will constitute a quorum. The membership consists of persons independent of the Principal Investigator who have no financial, scientific, or other conflict of interest with the trial. Current or past collaborators of Principal Investigator are not eligible to serve on the DSMC. Members of the DSMC will have clinical/content expertise in acute kidney injury; clinical trial methodology and/or biostatistics. The DSMC will meet twice per year during the trial.

### Ethical Considerations

The study has been reviewed and approved by the Health Research Ethics Board at the University of Alberta (File # 7362) (Additional File 1).

### Potential Challenges with the Trial

Research in patients with AKI has been traditionally challenging due to lack of a standardized definition or classification scheme for AKI. In order to address this issue, and optimize the potential generalizability, we have incorporated the RIFLE definition/classification system for AKI[42]. This consensus definition has now been validated and its clinical use is strengthened by its objectivity, simplicity and by the incorporation of a system to categorize the changes in severity of AKI over time.

The development of acute pulmonary edema in an ICU patient with AKI was identified in our survey as an indication for urgent furosemide or RRT and as such a potential barrier to compliance with our trial[20]. We have addressed this in our protocol by including a provision for the urgent use of furosemide in any enrolled patient with pulmonary edema at the discretion of the consultant ICU physician. Similarly, RRT can be initiated at any time.

Another potential issue with the protocol was the handling of an excess diuresis following initiation of the study drug. To address this, we have incorporated into our study algorithm provisions to temporarily discontinue the study drug in the event of an excess diuresis. Similarly, electrolyte abnormalities, in particular low magnesium and potassium, are common with use of furosemide. This issue is addressed by our ICU having standardized protocols for the replacement of both of these electrolytes in the event they are significantly low.

We do not anticipate any losses to follow-up during the study intervention. This is due to our primary outcome, progression in severity of kidney injury in AKI, being measured while the patient is admitted to ICU. A situation may arise where a patient is randomized and allocated as non-septic but subsequently develops sepsis during the trial. We will address this potential issue by analyzing all these patients according to their initial allocated group.

## Discussion

Furosemide continues to be commonly used in critically ill patients with AKI despite conflicting data on its efficacy and safety from clinical studies. This observation implies there is a misalignment between available evidence and clinical practice. Moreover, this suggests there is clinical equipoise for and there is an urgent need to generate higher-quality evidence on the safety and efficacy of furosemide from randomized trials to guide on this issue. This protocol, for a phase II randomized, blinded, placebo-controlled trial of a low-dose continuous infusion of furosemide, titrated to the physiology parameter of urine output, in critically ill patients early onset AKI, proposes to inform on this issue and potentially aid in the development, design and conduct of a phase III trial powered to evaluate a clinically relevant outcome such as renal replacement therapy or mortality.

## Competing interests

The authors declare that they have no competing interests.

## Authors' contributions

SMB, FAM, RB designed the trial; SMB, RTNG, FAM, RB obtained funding for the trial; SMB drafted the manuscript; SMB, RTNG, FAM, RB provided critical revision of the manuscript. All authors read and approved the final manuscript.

## Supplementary Material

Additional file 1 Ethics Approval forms - SPARK Study HREB and CH ethics approval forms. Click here for file

Additional file 2 Summary of SPARK Study profile on the Albert Innovates - Health Solutions (formerly the Alberta Heritage Foundation for Medical Research) website. Click here for file

Additional file 3 Summary of SPARK Study funding from Albert Innovates - Health Solutions (formerly the Alberta Heritage Foundation for Medical Research). Click here for file
