# Supplementary material for: The SPARK Study: a phase II randomized blinded controlled trial of the effect of furosemide in critically ill patients with early acute kidney injury
Source: Trials. 2010 May 11;11:50. doi: 10.1186/1745-6215-11-50 (PMC2874544; doi:10.1186/1745-6215-11-50)
Supplement: Additional file 1 — Ethics Approval forms - SPARK Study HREB and CH ethics approval forms. [file 1745-6215-11-50-S1.PDF]

Health Research Ethics Board

213 Heritage Medical Research Centre
University of Alberta, Edmonton, Alberta T6G 2S2
p.780.492.9724 (Biomedical Panel)
p.780.492.0302 (Health Panel)
p.780.492.0459
p.780.492.0839
f.780.492.7808

ETHICS APPROVAL FORM

Date of HREB Meeting: **April 25, 2008**

Name of Principal Investigator(s): **Dr. Sean Bagshaw**

Department: **Critical Care Medicine**

Title: **A phase II randomized blinded controlled trial of the effect of furosemide in critically ill patients with early acute kidney injury**

The Health Research Ethics Board (Biomedical Panel) has reviewed the protocol involved in this project which has been found to be acceptable within the limitations of human experimentation. The REB has also reviewed and approved the subject information material and consent form.

The Research Ethics Board assessed all matters required by section 50(1)(a) of the Health Information Act. Subject consent for access to identifiable health information is required for the research described in the ethics application, and appropriate procedures for such consent have been approved by the REB Panel.

Specific Comments:

JUL - 8 2008

S.K.M. Kimber, M.D., FRCPC
Chair of Health Research Ethics Board
Biomedical Panel

Date of approval release

This approval is valid for one year

Issue #7362

Capital Health
EDMONTON AREA

Regional Research Administration
Northern Alberta Clinical Trials and Research Centre
1800 College Plaza
8215 - 112 Street NW
Edmonton, AB T6G 2C8

ADMINISTRATIVE APPROVAL FOR PROPOSED RESEARCH

Operational Approvals:
UAH - ICU (General Systems)/ Critical Care

Protocol Number:

Protocol Acronym:

Protocol Title:
A Phase II Randomized Blinded Controlled Trial of the Effect of Furosemide in Critically Ill patients with Early Acute Kidney Injury

Principal Investigator: Dr. Sean Bagshaw

Faculty: Medicine & Dentistry

Department: Division of Critical Care Medicine

Division:

Research Locations:
University of Alberta Hospital

Sponsor: AHFMR (Alberta Heritage Foundation for Medical Research)

CRO:

Funding Type: Grant

O/H Rate - GST: 0% - 0%

Oracle Account:

Legacy Account:

Comments:

**Regional Research Admin File:**	6270
**Ethics File#:**	7362
**Ethics Approval Date:**	Jul 08, 2008
**Contract Finalized Date:**	
**Project Approval Date:**	Jul 21, 2008
**Approval Printed:**	Jul 21, 2008

July 22/08

Carlos Miranda
Regional Research Administration
Copy to Finance and Administration
