# Supplementary material for: The SPARK Study: a phase II randomized blinded controlled trial of the effect of furosemide in critically ill patients with early acute kidney injury
Source: Trials. 2010 May 11;11:50. doi: 10.1186/1745-6215-11-50 (PMC2874544; doi:10.1186/1745-6215-11-50)

[**Alberta Innovates- Health Solutions - Alberta Heritage Foundation for Medical Research**](http://www.ahfmr.ab.ca/)


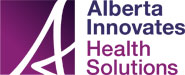


- [About Us](http://www.ahfmr.ab.ca/about.php)
- [Publications](http://www.ahfmr.ab.ca/publications/)
- [Experts](http://www.ahfmr.ab.ca/experts/search.php)
- [Programs](http://www.ahfmr.ab.ca/programs.php)
- [Media Desk](http://www.ahfmr.ab.ca/news.php)
- [Contact](http://www.ahfmr.ab.ca/contacts.php)

**Experts Database**

[Search](http://www.ahfmr.ab.ca/experts/search.php) | [Browse](http://www.ahfmr.ab.ca/experts/browse.php)

**Information for *Dr. Sean Bagshaw***:

| **ID** |  | 451 |
| --- | --- | --- |
| **Name** |  | **Dr. Sean Bagshaw** |
| **Department** |  | Division of Critical Care Medicine |
| **Faculty** |  | Medicine & Dentistry |
| **Institution** |  | University of Alberta |
| **Project Title** |  | A Randomized, Double-blind, Placebo-controlled Trial of the Effect of Furosemide in Critically Ill Patients with Early Acute Kidney Injury |
| **Description of Project** |  | Acute kidney injury-sometimes called acute renal failure-is a common complication experienced by patients in intensive care units. Dr. Sean Bagshaw is assessing a drug called furosemide for its ability to prevent progression, limit severity, and promote recovery from early acute kidney injury. |
| **Phone Number** |  | (780) 407-6755 |
| **Fax Number** |  | (780) 407-1228 |
| **E-mail** |  | [bagshaw@ualberta.ca](mailto:bagshaw@ualberta.ca) |
| **Website** |  |  |
| **AHFMR Publications** |  | |  | | --- | | None | |
| **Award Category** |  | Clinical Investigator |

© 2010 Alberta Innovates - Health Solutions builds on the strengths and successes of the former Alberta Heritage Foundation for Medical Research.


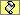

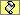

Supplement: Additional file 2 — Summary of SPARK Study profile on the Albert Innovates - Health Solutions (formerly the Alberta Heritage Foundation for Medical Research) website. [file 1745-6215-11-50-S2.DOC]
