# Supplementary material for: The SPARK Study: a phase II randomized blinded controlled trial of the effect of furosemide in critically ill patients with early acute kidney injury
Source: Trials. 2010 May 11;11:50. doi: 10.1186/1745-6215-11-50 (PMC2874544; doi:10.1186/1745-6215-11-50)

[**Alberta Innovates- Health Solutions - Alberta Heritage Foundation for Medical Research**](http://www.ahfmr.ab.ca/)


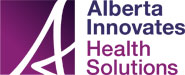


- [About Us](http://www.ahfmr.ab.ca/about.php)
- [Publications](http://www.ahfmr.ab.ca/publications/)
- [Experts](http://www.ahfmr.ab.ca/experts/search.php)
- [Programs](http://www.ahfmr.ab.ca/programs.php)
- [Media Desk](http://www.ahfmr.ab.ca/news.php)
- [Contact](http://www.ahfmr.ab.ca/contacts.php)

**Results of the September 2007 Independent Investigator Award Competitions**

AHFMR is delighted to announce the results of its September 2007 independent investigator award competitions. These awards mark the Foundation's transition to the [new awards structure](http://www.ahfmr.ab.ca/press/2006-09-14.php) announced in September 2006; AHFMR now offers the richest and longest health and medical research awards in Canada. The awards, offered to 54 internationally competitive researchers based in Alberta or in recruitment to the province, total more than $59 million. The majority of the awards are in excess of $1 million each.

Dr. Jacques Magnan, AHFMR Interim President & CEO, and the Foundation's Board of Trustees congratulate the successful applicants and acknowledge and thank all applicants for their substantial efforts.

**September 2007 competition awards (for implementation in July 2008)**

[Clinical Investigator](http://www.ahfmr.ab.ca/grants/awardlist2008.php#clinical)
[Scholar](http://www.ahfmr.ab.ca/grants/awardlist2008.php#scholar)
[Senior Scholar](http://www.ahfmr.ab.ca/grants/awardlist2008.php#seniorscholar)
[Scientist](http://www.ahfmr.ab.ca/grants/awardlist2008.php#scientist)
[Senior Investigator](http://www.ahfmr.ab.ca/grants/awardlist2008.php#investigator)
[Population Health Investigator](http://www.ahfmr.ab.ca/grants/awardlist2008.php#population)
[Health Scholar](http://www.ahfmr.ab.ca/grants/awardlist2008.php#healthscholar)
[Health Senior Scholar](http://www.ahfmr.ab.ca/grants/awardlist2008.php#seniorhealthscholar)
[Health Scientist](http://www.ahfmr.ab.ca/grants/awardlist2008.php#healthscientist)

**Applications**

In September 2007 AHFMR received 125 applications for the independent investigator awards announced below. These awards provide investigator support in the following categories: Clinical Investigator, Population Health Investigator, Scholar, Health Scholar, Senior Scholar, Health Senior Scholar, Scientist, and Health Scientist. One request was made for a Senior Investigator award.

**Assessments and Decisions**

All applications were assessed by [peer review committees](http://www.ahfmr.ab.ca/grants/committees.php) assisted by external reviews obtained from the broader scientific community. AHFMR wishes to acknowledge the substantial assistance provided by its committee members and external reviewers and their important ongoing contributions to the Foundation's mission.

Based on recommendations from the advisory committees, the [AHFMR Board of Trustees](http://www.ahfmr.ab.ca/about.php#trustees) made the final decisions regarding funding at their quarterly meeting on March 5, 2008.

Formal letters of offer will be sent to each university the week of March 24. These letters will include details on the amount of funding to be provided for the various components of the awards, including for establishment grants (where applicable). The reviews received for each application will also be provided. The list of successful applicants is provided for information only. The formal letters will serve as the Foundation's official decision should there be any discrepancy with the material on the web site.

**Results**

General competition statistics are provided [here](http://www.ahfmr.ab.ca/grants/results/AHFMR Personnel 200709 Competition Results Short Summary.pdf). The names of the successful candidates by program are provided below. Please note that the health awards are listed separately from the (bio)medical awards and that there may be subcategories within each award category (e.g. new Scholars are listed separately from Clinical Investigators progressing to the Scholar level).

**Advertising**

These awards will be advertised in the *National Post, Calgary Herald, Lethbridge Herald, Medicine Hat News, Red Deer Advocate, Grande Prairie Daily Herald, and Fort McMurray Today* on Thursday, March 13, 2008; Globe and Mail, Edmonton Journal, and Lloydminster Meridian Booster on Friday, March 14, 2008. The advertisements will also appear in the *Drumheller Mail* on March 19,*U of C Gauntlet* on March 27, *U of C On Campus* on April 4, *U of A Folio* on March 28, *U of A Gateway* on April 3, and the *U of L Legend* in April 2008.

**Clinical Investigator (New)**

[[Top]](http://www.ahfmr.ab.ca/grants/awardlist2008.php#top)

| **Candidate** | **Department** | **Faculty** | **Institution** | **Project Title** |
| --- | --- | --- | --- | --- |
| Bagshaw, Sean | Division of Critical Care Medicine | Medicine & Dentistry | University of Alberta | A Randomized, Double-blind, Placebo-controlled Trial of the Effect of Furosemide in Critically Ill Patients with Early Acute Kidney Injury |
| Butcher, Ken S. | Medicine | Medicine & Dentistry | University of Alberta | Cerebral Perfusion and Blood Pressure in Intracerebral Hemorrhage: A Safety Study |
| Oudit, Gavin Y. | Medicine | Medicine & Dentistry | University of Alberta | Role of Angiotensin Converting Enzyme 2 (ACE2) in Heart Disease: Using Human Recombinant ACE2 as Potential Therapy |
| Zygun, David A. | Critical Care Medicine | Medicine | University of Calgary | Proteomics of Severe Traumatic Brain Injury: Matrix Metalloproteinase Expression |

**Clinical Investigator (Renewal)**

[[Top]](http://www.ahfmr.ab.ca/grants/awardlist2008.php#top)

| **Candidate** | **Department** | **Faculty** | **Institution** | **Project Title** |
| --- | --- | --- | --- | --- |
| Barber, Philip A. | Clinical Neurosciences | Medicine | University of Calgary | Magnetic Resonance Molecular Imaging (MRMI) of the Early Endothelial Inflammatory Response in Focal Ischemia |
| Lehmann, Ordan | Ophthalmology | Medicine & Dentistry | University of Alberta | Copy Number Variation in Ocular Development and Glaucoma |
| Myers, Robert P. | Medicine | Medicine | University of Calgary | Optimizing the Management of Patients with Fibrotic Liver Diseases |
| Reiman, Anthony J. | Oncology | Medicine & Dentistry | University of Alberta | Therapeutic Targets in the Multiple Myeloma Centrosome |

**Scholarship (New)**

[[Top]](http://www.ahfmr.ab.ca/grants/awardlist2008.php#top)

| **Candidate** | **Department** | **Faculty** | **Institution** | **Project Title** |
| --- | --- | --- | --- | --- |
| Baksh, Shairaz | Pediatrics | Medicine & Dentistry | University of Alberta | The Role of Tumor Suppressor Protein, RASSF1A, in TNFalpha Signaling |
| Cameron, Lisa E. | Medicine | Medicine & Dentistry | University of Alberta | Molecular Regulation of CRTh2 and its Role in the Development of Allergy |
| Cobb, John A. | Biological Sciences | Science | University of Calgary | The Function and Regulation of Short-stature Genes during Embryonic Development |
| Grewal, Savraj | Biochemistry & Molecular Biology | Medicine | University of Calgary | Ribosome Synthesis and Cell Growth Control |
| Leslie, Elaine M. | Physiology | Medicine & Dentistry | University of Alberta | The Role of Phase II Metabolism and MRP Transport in Carcinogen Detoxification |
| Noskov, Sergei Y. | Biological Sciences | Science | University of Calgary | Computational Studies of Transport Proteins and Ion Channels |
| Touret, Nicolas | Biochemistry | Medicine & Dentistry | University of Alberta | Cell Biology of the Immune Receptor Dectin-1 |
| Yates, Robin M. |  | Veterinary Medicine | University of Calgary | Functional Intraphagosomal Dynamics in the Macrophage |

**Scholar (Clinical Investigator to Scholar)**

[[Top]](http://www.ahfmr.ab.ca/grants/awardlist2008.php#top)

No Awards

**Senior Scholar (New)**

[[Top]](http://www.ahfmr.ab.ca/grants/awardlist2008.php#top)

| **Candidate** | **Department** | **Faculty** | **Institution** | **Project Title** |
| --- | --- | --- | --- | --- |
| Sauve, Yves | Ophthalmology | Medicine & Dentistry | University of Alberta | Novel Approaches to Study and Prevent Vision Loss |
| Zheng, Xi-Long | Biochemistry & Molecular Biology | Medicine | University of Calgary | Regulation of Myocardin Expression in Vascular Smooth Muscle: Relevance to Cardiovascular Diseases |

**Senior Scholar (Scholar to Senior Scholar)**

[[Top]](http://www.ahfmr.ab.ca/grants/awardlist2008.php#top)

| **Candidate** | **Department** | **Faculty** | **Institution** | **Project Title** |
| --- | --- | --- | --- | --- |
| Childs, Sarah J. | Biochemistry & Molecular Biology | Medicine | University of Calgary | Signaling in Vascular Development |
| Dickson, Clayton T. | Psychology | Science | University of Alberta | Coordination of Sleep-like Forebrain Rhythms in Anaesthesia |
| Mason, Andrew L. | Medicine | Medicine & Dentistry | University of Alberta | Retroviral Induction of Autoimmune Liver Disease |
| Metz, Gerlinde A. | Neuroscience | Arts & Science | University of Lethbridge | The Impact of Stress on Motor System Function and Disease |
| Raivio, Tracy L. | Biological Sciences | Science | University of Alberta | Genetic Dissection of Enteropathogenic Escherichia coli Virulence |
| Salo, Paul T. | Surgery | Medicine | University of Calgary | Mechanisms of Neurovascular Interactions in Connective Tissue Injury and Repair |
| Simmonds, Andrew J. | Cell Biology | Medicine & Dentistry | University of Alberta | Dissecting the Functional Roles of RNA Based Regulation during Embryonic Development and Differentiation |
| Yan, Jun | Physiology & Biophysics | Medicine | University of Calgary | Cholinergic Mechanisms for Early Development and Learning-Induced Neural Plasticity of the Auditory Cortex: Role of Muscarinic Acetylcholine Receptor Subtypes |

**Scientist (New)**

[[Top]](http://www.ahfmr.ab.ca/grants/awardlist2008.php#top)

| **Candidate** | **Department** | **Faculty** | **Institution** | **Project Title** |
| --- | --- | --- | --- | --- |
| Giles, Wayne R. |  | Kinesiology | University of Calgary | Cell-Cell Communication in the Mammalian Heart: Fibroblast, Myofibroblast, Myocyte Interactions |
| Gill, Ronald G. | Medical Microbiology & Immunology | Medicine & Dentistry | University of Alberta | The Role of Natural Killer (NK) Cells in Transplantation Tolerance |

**Scientist (Senior Scholar to Scientist)**

[[Top]](http://www.ahfmr.ab.ca/grants/awardlist2008.php#top)

| **Candidate** | **Department** | **Faculty** | **Institution** | **Project Title** |
| --- | --- | --- | --- | --- |
| Funk, Gregory D. | Physiology | Medicine & Dentistry | University of Alberta | Deciphering the Differential Modulation of Respiratory Rhythm Generating and Pattern Forming Networks by ATP |
| McFarlane, Sarah | Cell Biology & Anatomy | Medicine | University of Calgary | Neuronal Morphogenesis in the Visual System |
| Nguyen, Peter V. | Physiology | Medicine & Dentistry | University of Alberta | Noradrenergic Regulation of Synaptic Plasticity and Memory |
| Patel, Kamala D. | Physiology & Biophysics | Medicine | University of Calgary | Mechanotransduction in Leukocyte Trafficking |

**Scientist (Renewal)**

[[Top]](http://www.ahfmr.ab.ca/grants/awardlist2008.php#top)

| **Candidate** | **Department** | **Faculty** | **Institution** | **Project Title** |
| --- | --- | --- | --- | --- |
| Ballanyi, Klaus | Physiology | Medicine & Dentistry | University of Alberta | Metabolic Control of Neonatal Breathing |
| Greer, John J. | Physiology | Medicine & Dentistry | University of Alberta | Respiratory Control and Development |
| McGhee, James D. | Biochemistry & Molecular Biology | Medicine | University of Calgary | Development of the C. elegans Intestine |
| Santamaria, Pere | Microbiology & Infectious Diseases | Medicine | University of Calgary | A Novel Vaccine for the Prevention and Cure of Type 1 Diabetes |
| Turner, Raymond W. | Cell Biology & Anatomy | Medicine | University of Calgary | Ionic and Synaptic Control of Cerebellar Output |
| Weiss, Samuel | Cell Biology & Anatomy | Medicine | University of Calgary | Neurogenesis by Central Nervous System Stem Cells |

**Senior Investigator**

[[Top]](http://www.ahfmr.ab.ca/grants/awardlist2008.php#top)

| **Candidate** | **Department** | **Faculty** | **Institution** | **Project Title** |
| --- | --- | --- | --- | --- |
| Moqbel, Redwan | Medicine | Medicine & Dentistry | University of Alberta | Intracellular Mechanisms Regulating Exocytosis and Mediator Release in Human Eosinophils |

**Population Health Investigator (New)**

[[Top]](http://www.ahfmr.ab.ca/grants/awardlist2008.php#top)

| **Candidate** | **Department** | **Faculty** | **Institution** | **Project Title** |
| --- | --- | --- | --- | --- |
| Colman, Ian R. | Public Health Sciences | School of Public Health | University of Alberta | Early-life Neurodevelopment, Stressful Life Events and Depression Across the Life Course |
| Eurich, Dean T. | Public Health Sciences | School of Public Health | University of Alberta | PHANTM (Patients with Heart Failure and Type 2 Diabetes Treated with Metformin) Study |
| Ferber, (Ronald) Reed |  | Kinesiology | University of Calgary | The Relationship Between Patellofemoral Pain Syndrome, Gait Biomechanics, and Muscular Strength |
| Jette, Nathalie | Clinical Neurosciences | Medicine | University of Calgary | The Development of an Appropriateness and Necessity Rating Tool to Identify Patient with Potentially Resectable Focal Epilepsy |
| Rempel, Gwendolyn R. |  | Nursing | University of Alberta | Safeguarding Precarious Survival: Building Capacity in Parents of Children with Complex Congenital Heart Disease |

**Population Health Investigator (Renewal)**

[[Top]](http://www.ahfmr.ab.ca/grants/awardlist2008.php#top)

| **Candidate** | **Department** | **Faculty** | **Institution** | **Project Title** |
| --- | --- | --- | --- | --- |
| Ball, Geoff D. C. | Pediatrics | Medicine & Dentistry | University of Alberta | The PAC Study: Using Parents as Agents of Change in Pediatric Weight Management |
| Burstyn, Igor | Medicine | Medicine & Dentistry | University of Alberta | Maternal Exposure to Common Pollutants and Fetal Growth Restriction: Consequences and Interventions |
| Hemmelgarn, Brenda | Medicine | Medicine | University of Calgary | Progression and Management of Chronic Kidney Disease Among First Nations People |

**Health Scholarship Competition (New)**

[[Top]](http://www.ahfmr.ab.ca/grants/awardlist2008.php#top)

| **Candidate** | **Department** | **Faculty** | **Institution** | **Project Title** |
| --- | --- | --- | --- | --- |
| Carlson, Linda E. | Oncology | Medicine | University of Calgary | Evaluation of Online Distress Screening for Newly Diagnosed Cancer Patients: A Randomized Controlled Trial |

**Health Scholarship Competition (Population Health Investigator to Health Scholar)**

[[Top]](http://www.ahfmr.ab.ca/grants/awardlist2008.php#top)

| **Candidate** | **Department** | **Faculty** | **Institution** | **Project Title** |
| --- | --- | --- | --- | --- |
| King, Kathryn M. |  | Nursing | University of Calgary | Determinants of Cardiovascular Health Decision Making: A Mixed Methods Program of Research |
| Vohra, Sunita | Pediatrics | Medicine & Dentistry | University of Alberta | Active Surveillance to Access the Safety of Common Complementary and Alternative Medicine (CAM) Therapies |

**Health Senior Scholar Competition (New)**

[[Top]](http://www.ahfmr.ab.ca/grants/awardlist2008.php#top)

| **Candidate** | **Department** | **Faculty** | **Institution** | **Project Title** |
| --- | --- | --- | --- | --- |
| Goodman, Karen J. | Medicine | Medicine & Dentistry | University of Alberta | H. pylori Infection in Northwest Territories Aboriginal Communities |
| Lee, Shoo K. | Pediatrics | Medicine & Dentistry | University of Alberta | A Program for Knowledge Translation and Health Care Improvement |

**Health Senior Scholarship Competition (Health Scholar to Health Senior Scholar)**

[[Top]](http://www.ahfmr.ab.ca/grants/awardlist2008.php#top)

| **Candidate** | **Department** | **Faculty** | **Institution** | **Project Title** |
| --- | --- | --- | --- | --- |
| Carroll, Linda J. | Public Health Sciences | School of Public Health | University of Alberta | Moving from Prognosis to Intervention: Mapping the Pathways to Recovery from Musculoskeletal Injury |
| Friedenreich, Christine M. | Population Health & Information |  | Alberta Cancer Board | Physical Activity in Cancer Control: From Observational to Intervention Research |

**Health Scientist**

[[Top]](http://www.ahfmr.ab.ca/grants/awardlist2008.php#top)

No Applications Received

© 2010 Alberta Innovates - Health Solutions builds on the strengths and successes of the former Alberta Heritage Foundation for Medical Research.


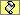

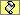

Supplement: Additional file 3 — Summary of SPARK Study funding from Albert Innovates - Health Solutions (formerly the Alberta Heritage Foundation for Medical Research). [file 1745-6215-11-50-S3.DOC]
